# High-Intensity Acute Exercise and Directed Forgetting on Memory Function

**DOI:** 10.3390/medicina55080446

**Published:** 2019-08-07

**Authors:** Mary Elizabeth Pace, Paul D. Loprinzi

**Affiliations:** Exercise & Memory Laboratory, Department of Health, Exercise Science and Recreation Management, The University of Mississippi, Oxford, MS 38677, USA

**Keywords:** episodic memory, executive control, prefrontal cortex, physical activity

## Abstract

*Background and Objectives*: Despite accumulating research demonstrating that acute exercise may enhance memory function, very little research has evaluated whether acute exercise can effectuate intentional directed forgetting (DF), an adaptative strategy to facilitate subsequent memory performance.* Materials and Methods:* A three-arm parallel-group randomized controlled intervention was employed. Participants were randomized into one of three groups, including: (1) exercise plus DF (Ex + DF), (2) DF (directed forgetting) only (DF) and (3) R (remember) only (R). The acute bout of exercise included 15 min of high-intensity treadmill exercise. The memory assessment involved the presentation of two-word lists. After encoding the first word list, participants were either instructed to forget all of those words (DF) or to remember them. Following this, participants encoded the second word list. *Results:* We observed a statistically significant main effect for list *F*(1, 57) = 12.27, *p* < 0.001, η^2^_p_ = 0.18, but no main effect for group *F*(2, 57) = 1.32, *p* = 0.27, η^2^_p_ = 0.04, or list by group interaction, *F*(2, 57) = 2.89, *p* = 0.06, η^2^_p_ = 0.09. *Conclusions*: This study demonstrates a directed forgetting effect in that cueing an individual to forget a previously encoded list of items facilitates memory performance on a subsequent list of items. However, we failed to demonstrate any beneficial effect of acute exercise in facilitating directed forgetting. These findings are discussed in the context of directed forgetting theories, particularly the attention inhibition mechanism, as well as the timing of the acute bout of exercise.

## 1. Introduction

Optimal memory function is critical for daily functioning—we use our past memories to help shape our current and future behaviors. As such, identifying ways to enhance memory function is of great importance. We have extensively demonstrated that acute exercise [1,2], particularly high-intensity exercise [3], can enhance memory function, including episodic memory [4,5] and working memory capacity [6]. Mechanisms of this effect can be found elsewhere [7,8,9,10,11], and include, for example, increased neuronal excitability in key memory-related brain structures (e.g., hippocampus) [12]. What has been considerably less investigated, however, is whether acute exercise can effectuate intentional forgetting. Intentional forgetting, as opposed to unintentional forgetting, is considered a productive, adaptative skill. Memories are forgotten for several reasons, including, for example, conflicting information being similar in context [13]. As such, intentionally forgetting certain material may minimize a future memory interference effect. As a real-world example, an individual may be studying for an upcoming examination, and as such, they may be encoding large amounts of information. They may eventually realize that some of this information may not be necessary to remember (either determined by themselves or instructed by the teacher). Intentionally forgetting some of this previously encoded information may help to consolidate other learned information, as well as to facilitate the encoding of subsequently acquired information. 

Although acute exercise has been shown to enhance the acquisition and retention of information [1,2], to our knowledge, only one study has evaluated the effects of acute exercise on intentional forgetting [14]. In a previous experiment [14], we evaluated a selective directed forgetting paradigm. That is, participants encoded a list of words (List 1) and were subsequently asked to either selectively forget some of the List 1 words (selective forgetting), forget all of the List 1 words (directed forgetting), or remember all of the List 1 words (remember). They then encoded a separate list of words (List 2). Generally, participants instructed to forget information (List 1) tended to recall more words on List 2 (i.e., a List 1 forgetting-induced List 2 enhancement effect). Previous research suggested mixed findings regarding whether individuals can selectively forget partial information [15]. As such, in this previous experiment [14], in addition to these three groups (selective forgetting, directed forgetting, and remember), we employed a condition that combined selective forgetting with an acute bout of high-intensity exercise. Given the role of executive function in facilitating selective forgetting [15], coupled with the executive function enhancement effects of acute exercise [16], we hypothesized a List 2 enhancement effect in this group, over and above that of the group that received the selective forgetting instructions. Although we did observe a List 2 enhancement effect from directed forgetting, acute exercise did not have any effect on selective forgetting [14]. 

The current experiment extends this past experiment in two main ways. First, in this experiment, we aimed to replicate this directed forgetting effect with novelty added to this paradigm; this experiment evaluates whether acute exercise can augment the List 2 enhancement effect from directed forgetting. A second extension of our past experiment [14] was to employ a higher-intensity bout of exercise. Emerging work suggests that high-intensity acute exercise is superior in enhancing episodic memory when compared to lower-intensity acute exercise [3,17]. The null exercise effect in our past experiment [14] may have been a result of the lower-intensity (i.e., moderate-intensity) exercise protocol that we employed. Thus, the purpose of the present experiment was to evaluate whether we can replicate a directed forgetting effect and whether high-intensity acute exercise can additively enhance a directed forgetting effect. The implication of this work stems from the adaptative benefits of intentional forgetting. Thus, given that it is not realistic to remember all previously acquired information, coupled with the notion that intentional forgetting of select information may help to facilitate the recall of other select information as well as enhance the encoding of subsequent information, it is advantageous to identify strategies to facilitate intentional forgetting. The strategy of interest for this experiment was acute exercise. We hypothesized that acute exercise would enhance an intentional forgetting effect. If such an effect is observed, then it may be sensible for individuals to consider engaging in a brief bout of acute exercise prior to learning, which at that point, they may engage in intentional forgetting processes if encoding concurrent, conflicting/unnecessary stimuli. 

## 2. Materials & Methods

### 2.1. Study Design

A three-arm parallel-group randomized controlled intervention was employed. Participants were randomized into one of three groups, including two experimental groups and a control group. One of the experimental groups engaged in a bout of high-intensity acute exercise for 15 min (treadmill jogging), while the other two groups did not exercise. The three groups included: (1) exercise plus DF (Ex + DF), (2) DF (directed forgetting) only (DF) and (3) R (remember) only (R). Details on the procedures of these groups is explained in the protocol below. Participants provided written consent prior to participation. This study was approved by the ethics committee at the University of Mississippi (#19-041).

### 2.2. Participants

Each group included 20 participants (*N* = 60; *n* = 20 per group), which aligns with our other related experimental work on this topic [18,19,20,21]. Participants were randomized into one of three groups using a computer-generated program. Recruitment occurred via a convenience-based, non-probability sampling approach (classroom announcement and word-of-mouth). Participants included undergraduate and graduate students between the ages of 18 and 40 years.

Additionally, participants were excluded if they: self-reported as a daily smoker [22,23], self-reported being pregnant [24], exercised within 5 h of testing [25], consumed caffeine within 3 h of testing [26], had a concussion or head trauma within the past 30 days [27], took marijuana or other memory-altering substances within the past 30 days [28], or were considered a daily alcohol user (>30 drinks/month for women; >60 drinks/month for men) [29].

### 2.3. Exercise Protocol

Those randomized to the exercise group (Ex + DF) exercised at 80% of their heart rate reserve (with maximum heart rate estimated from the formula, 220-age), constituting high-intensity exercise [30]. The treadmill speed and incline were manipulated throughout the bout of exercise to ensure the heart rate (measured continuously via Polar, F1, chest-strapped monitor) stayed within 5 beats per minute of the target heart rate. Heart rate was monitored throughout the bout of exercise and recorded at baseline, midpoint (7.5 min) and endpoint (15-min). This manipulation of speed and incline resulted in an achieved exercise heart rate of 77.2% of heart rate reserve, which corresponds to vigorous-intensity exercise (60–89% of heart rate reserve) [30]. 

### 2.4. Non-Exercise Protocol

As stated above, the Ex + DF group engaged in an acute bout of exercise for 15 min and then rested for 10 min before starting the memory protocol. The two other groups (DF and R) engaged in a seated, time-matched 25-min task before starting the memory protocol. This involved playing a medium-level on-line administered Sudoku puzzle. The website for this puzzle is located here: https://www.websudoku.com/. We have experimental evidence that playing this puzzle does not prime or enhance memory function [31]. Similar to the exercise protocol, heart rate was measured and recorded at baseline, midpoint (12.5 min) and endpoint (25 min).

### 2.5. Memory Assessment

The memory assessment involved the presentation of two-word lists (list 1 and list 2; L1 and L2, respectively). Each list consisted of 16 unrelated words from the Toronto Word Pool (http://datavis.ca/papers/twp.pdf). The order of words on each list was random. Each word was presented on a computer screen at a rate of 4 s, followed by a 1-s inter-stimulus interval. 

Prior to L1 presentation, participants received the following instructions, “do your best to try and memorize all of these words, as later in this experiment you will be asked to recall as many of these words as possible.” Following L1 presentation, participants received specific instructions, depending on the group they were randomized into. 

Those randomized into the DF (directed forgetting) group were told: “you now will be exposed to a different list of words in which you will be asked to recall later in this experiment. Importantly, please forget all the words from the previous list that you just saw. You will not be asked to remember any of these words from that previous list, and it is important to try and forget all those words, as forgetting those words will help you remember the words from the next task”.

Those randomized into the R (remember) group were told: “you will now be exposed to a different list of words in which you will be asked to recall later in this experiment. Please also do your best to remember all the words from the list you were just exposed to, as you will be asked to recall words from both lists”.

After the presentation of L2, participants completed simple arithmetic problems (for 30-s). Following this, participants were told: “you now will recall as many words as possible. You now have 90-s to recall as many words as you can remember from L1”. After this 90-s period, they were told: “You now will recall as many words as possible from L2”. Notably, the order of L1 and L2 were counterbalanced at both encoding and retrieval.

### 2.6. Statistical Analysis

All statistical analyses were computed in JASP (v. 0.9.2.0; Amsterdam, The Netherlands). A two-factor mixed-measures ANOVA was employed. Specifically, a 2 (List 1, List 2) × 3 (Ex + DF, DF, R) ANOVA was computed. Statistical significance was established as an alpha of 0.05. Partial eta-squared (η^2^_p_) values were calculated as a measure of effect size.

## 3. Results

Demographic Characteristics 

Table 1 displays the demographic characteristics of the sample. Participants were similar across the three groups.

Table 2 displays the heart rate response to the experimental conditions. There was a statistically significant main effect for time, *F*(2, 112) = 389.5, *p* < 0.001, η^2^_p_ = 0.87, main effect for condition, *F*(2, 56) = 215.9, *p* < 0.001, η^2^_p_ = 0.89, and a significant time by condition interaction, *F*(4, 112) = 351.5, *p* < 0.001, η^2^_p_ = 0.93.

**Figure 1:**
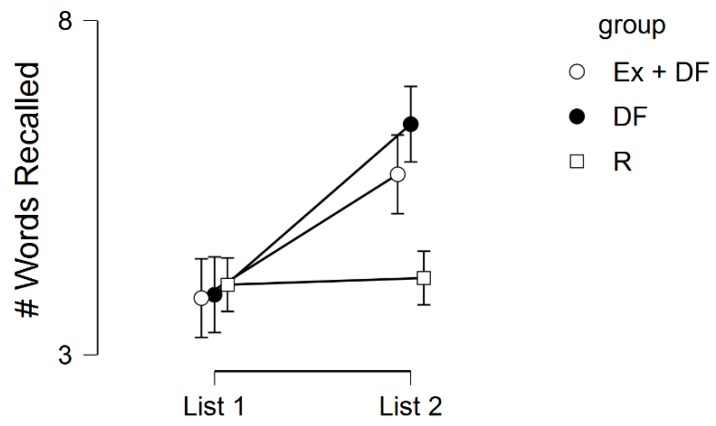
Memory performances across the experimental conditions. Error bars represent standard errors. DF, Directed Forgetting; Ex, Exercise; R, Remember; #, Number.

Table 3 and Figure 1 display the memory scores across the three experimental conditions. There was a statistically significant main effect for list, *F*(1, 57) = 12.27, *p* < 0.001, η^2^_p_ = 0.18, but no main effect for group, *F*(2, 57) = 1.32, *p* = 0.27, η^2^_p_ = 0.04, or list by group interaction, *F*(2, 57) = 2.89, *p* = 0.06, η^2^_p_ = 0.09. Regarding the list effect, Bonferroni-corrected post-hoc tests indicated that List 1 recall was significantly lower than List 2 recall, *M*_diff_ = −1.50, *p* = 0.001. For List 1, there were no differences across any of the groups, all *p*’s > 0.30. For List 2, Ex + DF was not different than DF, *M*_diff_ = −0.75, *p* = 0.44, but was just outside the significance threshold when compared to R, *M*_diff_ = 1.55, *p* = 0.06. For List 2, DF was different than R, *M*_diff_ = 2.3, *p* = 0.01.

## 4. Discussion

To date, only one study has evaluated whether acute exercise can effectuate intentional forgetting [14]. This is theoretically plausible, as intentional forgetting appears to be heavily influenced by executive control [32], with executive control shown to be enhanced with both acute moderate-intensity [33,34] and high-intensity [35,36] exercise. Further, intentional forgetting has been shown to activate prefrontal cortices associated with cognitive control (e.g., cingulate cortex), as well as induce the activation of the medial temporal lobe [37], both of which have been shown to become activated with acute exercise [38]. In our past experiment [14] and in the present study, we demonstrated that directed forgetting enhances subsequent memory performance. However, in both of our studies, we failed to demonstrate any beneficial effect of acute exercise in facilitating selective directed forgetting [14] or directed forgetting alone (present experiment). 

Intentional directed forgetting is thought to operate under active cognitive processes that engage attentional mechanisms, specifically active withdrawal of attention [39]. Thus, mobilization of attention via activation of the orienting attentional system is likely a key mechanism underlying intentional directed forgetting. Changes in internal context are also likely to play an important role in facilitating directed forgetting [40]. For example, in the remember group, they were instructed to remember both lists, and thus, they internally treated both lists as the same event, maintaining the internal context of the situation. However, those instructed to forget List 1 may have viewed it as a practice session, and as such, treated the two lists as separate events, inducing a mismatch in internal context, ultimately facilitating List 2 enhancement. Of course, many other theories of directed forgetting have been proposed and evaluated, including active erasure [41], selective rehearsal [42], and tagging and selective search [43].

In the context of acute exercise, the attention inhibition theory is likely the most relevant theory in which acute exercise would play any role. As stated, the attention inhibition theory suggests that an active process is used to reduce accessibility of the forgotten items in memory. At the point of encoding, when hearing the directed forgetting instruction, items are suppressed via an effortful inhibitory process. In the present experiment, as well as our previous experiment, we intentionally placed the acute bout of exercise prior to memory encoding in an effort to prime such executive control processes. However, future work should consider placing the acute bout of exercise immediately after the directed forgetting instruction. This may have a greater effect in priming such attention inhibition mechanisms and ultimately disrupting the retrieval paths for the forgetting items (List 1). 

## 5. Conclusions

In conclusion, we demonstrated a directed forgetting effect, in that cueing an individual to forget a previously encoded list facilitates memory performance on a subsequent list. Future work needs to continue to investigate the potential role, if any, that acute exercise plays in facilitating intentional forgetting. Such studies, for example, should consider the temporal effects of the acute bout of exercise on intentional forgetting.

## Figures and Tables

**Table 1:** Characteristics of the sample.

Variable	Ex + DF	DF	R
Age, mean years	20.2 (4.4)	20.9 (1.0)	21.0 (1.6)
Gender, % Female	45.0	65.0	65.0
Race-Ethnicity, % White	85.0	95.0	70.0
BMI, mean kg/m^2^	26.3 (4.4)	25.6 (6.2)	25.5 (4.4)

DF, Directed Forgetting; Ex, Exercise; R, Remember.

**Table 2:** Heart rate responses across the conditions (mean (sd)).

Heart Rate	Ex + DF	DF	R
Resting	79.6 (14.9)	74.8 (11.5)	73.3 (10.7)
Midpoint	154.7 (9.2)	74.6 (13.1)	75.9 (11.9)
Endpoint	172.4 (4.9)	74.7 (15.0)	76.2 (12.7)

DF, Directed Forgetting; Ex, Exercise; R, Remember.

**Table 3:** Memory scores (number of words recalled) across the conditions (mean (sd)).

List	Ex + DF	DF	R
List 1	3.85 (2.2)	3.90 (2.9)	4.05 (2.11)
List 2	5.70 (2.6)	6.45 (3.3)	4.15 (2.5)

DF, Directed Forgetting; Ex, Exercise; R, Remember.

## References

[1] Loprinzi P.D., Blough J., Crawford L., Ryu S., Zou L., Li H. (2019). The temporal effects of acute exercise on episodic memory function: Systematic review with meta-analysis. Brain Sci..

[2] Loprinzi P.D., Frith E., Edwards M.K., Sng E., Ashpole N. (2018). The effects of exercise on memory function among young to middle-aged adults: Systematic review and recommendations for future research. Am. J. Health Promot..

[3] Loprinzi P.D. (2018). Intensity-specific effects of acute exercise on human memory function: Considerations for the timing of exercise and the type of memory. Health Promot. Perspect..

[4] Sng E., Frith E., Loprinzi P.D. (2018). Experimental effects of acute exercise on episodic memory acquisition: Decomposition of multi-trial gains and losses. Physiol. Behav..

[5] Haynes J.T., Frith E., Sng E., Loprinzi P.D. (2018). Experimental effects of acute exercise on episodic memory function: Considerations for the timing of exercise. Psychol. Rep..

[6] Tillman B., Loprinzi P.D. (2019). The experimental effects of acute exercise intensity on episodic memory and working memory function. J. Neurobehav. Sci..

[7] Loprinzi P.D., Edwards M.K., Frith E. (2017). Potential avenues for exercise to activate episodic memory-related pathways: A narrative review. Eur. J. Neurosci..

[8] Loprinzi P.D. (2019). The role of astrocytes on the effects of exercise on episodic memory function. Physiol. Int..

[9] Loprinzi P.D., Frith E. (2019). A brief primer on the mediational role of BDNF in the exercise-memory link. Clin. Physiol. Funct. Imaging.

[10] Loprinzi P.D. (2019). Does brain-derived neurotrophic factor mediate the effects of exercise on memory?. Physician Sportsmed..

[11] Loprinzi P.D., Ponce P., Frith E. (2018). Hypothesized mechanisms through which acute exercise influences episodic memory. Physiol. Int..

[12] Loprinzi P.D. (2019). The effects of exercise on long-term potentiation: A candidate mechanism of the exercise-memory relationship. OBM Neurobiol..

[13] Davis R.L., Zhong Y. (2017). The biology of forgetting—A perspective. Neuron.

[14] Ferguson L., Cantrelle J., Loprinzi P.D. (2018). Experimental effects of exercise on forgetting. OBM Integr. Complement. Med..

[15] Aguirre C., Gomez-Ariza C.J., Andres P., Mazzoni G., Bajo M.T. (2017). Exploring mechanisms of selective directed forgetting. Front. Psychol..

[16] Chang Y.K., Labban J.D., Gapin J.I., Etnier J.L. (2012). The effects of acute exercise on cognitive performance: A meta-analysis. Brain Res..

[17] Crawford L., Loprinzi P.D. (2019). Effects of intensity-specific acute exercise on paired-associative memory and memory interference. Psych.

[18] Loprinzi P.D., Kane C.J. (2015). Exercise and cognitive function: A randomized controlled trial examining acute exercise and free-living physical activity and sedentary effects. Mayo Clin. Proc..

[19] Crush E.A., Loprinzi P.D. (2017). Dose-response effects of exercise duration and recovery on cognitive functioning. Percept. Mot. Sk..

[20] Frith E., Sng E., Loprinzi P.D. (2017). Randomized controlled trial evaluating the temporal effects of high-intensity exercise on learning, short-term and long-term memory, and prospective memory. Eur. J. Neurosci..

[21] Sng E., Frith E., Loprinzi P.D. (2018). Temporal effects of acute walking exercise on learning and memory. Am. J. Health Promot..

[22] Jubelt L.E., Barr R.S., Goff D.C., Logvinenko T., Weiss A.P., Evins A.E. (2008). Effects of transdermal nicotine on episodic memory in non-smokers with and without schizophrenia. Psychopharmacology.

[23] Klaming R., Annese J., Veltman D.J., Comijs H.C. (2016). Episodic memory function is affected by lifestyle factors: A 14-year follow-up study in an elderly population. Aging Neuropsychol. Cogn..

[24] Henry J.D., Rendell P.G. (2007). A review of the impact of pregnancy on memory function. J. Clin. Exp. Neuropsychol..

[25] Labban J.D., Etnier J.L. (2011). Effects of acute exercise on long-term memory. Res. Q. Exerc. Sport.

[26] Sherman S.M., Buckley T.P., Baena E., Ryan L. (2016). Caffeine enhances memory performance in young adults during their non-optimal time of day. Front. Psychol..

[27] Wammes J.D., Good T.J., Fernandes M.A. (2017). Autobiographical and episodic memory deficits in mild traumatic brain injury. Brain Cogn..

[28] Hindocha C., Freeman T.P., Xia J.X., Shaban N.D.C., Curran H.V. (2017). Acute memory and psychotomimetic effects of cannabis and tobacco both ‘joint’ and individually: A placebo-controlled trial. Psychol. Med..

[29] Le Berre A.P., Fama R., Sullivan E.V. (2017). Executive functions, memory, and social cognitive deficits and recovery in chronic alcoholism: A critical review to inform future research. Alcohol. Clin. Exp. Res..

[30] Garber C.E., Blissmer B., Deschenes M.R., Franklin B.A., Lamonte M.J., Lee I.M., Swain D.P. (2011). American College of Sports Medicine position stand. Quantity and quality of exercise for developing and maintaining cardiorespiratory, musculoskeletal, and neuromotor fitness in apparently healthy adults: Guidance for prescribing exercise. Med. Sci. Sports Exerc..

[31] Blough J., Loprinzi P.D. (2019). Experimental manipulation of psychological control scenarios: Implications for exercise and memory research. Psych.

[32] Anderson M.C., Green C. (2001). Suppressing unwanted memories by executive control. Nature.

[33] Hillman C.H., Snook E.M., Jerome G.J. (2003). Acute cardiovascular exercise and executive control function. Int. J. Psychophysiol..

[34] Ludyga S., Gerber M., Brand S., Holsboer-Trachsler E., Puhse U. (2016). Acute effects of moderate aerobic exercise on specific aspects of executive function in different age and fitness groups: A meta-analysis. Psychophysiology.

[35] Peruyero F., Zapata J., Pastor D., Cervello E. (2017). The acute effects of exercise intensity on inhibitory cognitive control in adolescents. Front. Psychol..

[36] Brown D., Bray S.R. (2018). Acute effects of continuous and high-intensity interval exercise on executive function. J. Appl. Biobehav. Res..

[37] Wylie G.R., Foxe J.J., Taylor T.L. (2008). Forgetting as an active process: An fMRI investigation of item-method-directed forgetting. Cereb. Cortex.

[38] Weng T.B., Pierce G.L., Darling W.G., Falk D., Magnotta V.A., Voss M.W. (2017). The acute effects of aerobic exercise on the functional connectivity of human brain networks. Brain Plast..

[39] Fawcett J.M., Taylor T.L. (2010). Directed forgetting shares mechanisms with attentional withdrawal but not with stop-signal inhibition. Mem. Cogn..

[40] Sahakyan L., Kelley C.M. (2002). A contextual change account of the directed forgetting effect. J. Exp. Psychology. Learn. Mem. Cogn..

[41] Basden B.H., Basden D.R., Gargano G.J. (1993). Directed forgetting in implicit and explicit memory tests: A comparison of methods. J. Exp. Psychol. Learn. Mem. Cogn..

[42] Wetzel C.D., Hunt R.E. (1977). Cue delay and the role of rehearsal in directed forgetting. J. Exp. Psychol. Hum. Learn. Mem..

[43] Epstein W., Massaro D.W., Wilder L. (1972). Selective search in directed forgetting. J. Exp. Psychol..

